# Sonodynamic therapy reduces iron retention of hemorrhagic plaque

**DOI:** 10.1002/btm2.10193

**Published:** 2020-10-31

**Authors:** Bicheng Li, Jie Gong, Siqi Sheng, Minqiao Lu, Shuyuan Guo, Jianting Yao, Haiyu Zhang, Xuezhu Zhao, Zhengyu Cao, Xin Sun, Huan Wang, Yang Cao, Yongxing Jiang, Zhen Tian, Bin Liu, Hua Zhao, Zhiguo Zhang, Hong Jin, Ye Tian

**Affiliations:** ^1^ Department of Cardiology, The First Affiliated Hospital, Cardiovascular Institute Harbin Medical University Harbin People's Republic of China; ^2^ Department of Pathophysiology and Key Laboratory of Cardiovascular Pathophysiology Key Laboratory of Cardiovascular Medicine Research (Harbin Medical University), Ministry of Education Harbin People's Republic of China; ^3^ Key Laboratory of Noise and Vibration Research, Institute of Acoustics Chinese Academy of Sciences Beijing People's Republic of China; ^4^ School of Materials and Engineering Harbin Institute of Technology Harbin People's Republic of China; ^5^ School of Instrumentation Science and Engineering Harbin Institute of Technology Harbin People's Republic of China; ^6^ Molecular Vascular Medicine, Medicine Department Karolinska University Hospital Solna Sweden

**Keywords:** ferroportin 1, intraplaque hemorrhage, iron, macrophage, sonodynamic therapy

## Abstract

Intraplaque hemorrhage (IPH) plays a major role in the aggressive progression of vulnerable plaque, leading to acute cardiovascular events. We previously demonstrated that sonodynamic therapy (SDT) inhibits atherosclerotic plaque progression. In this study, we investigated whether SDT could also be applied to treat more advanced hemorrhagic plaque and addressed the underlying mechanism. SDT decreased atherosclerotic burden, positively altered atherosclerotic lesion composition, and alleviated iron retention in rabbit hemorrhagic plaques. Furthermore, SDT reduced iron retention by stimulating *ferroportin 1 (Fpn1)* expression in *apolipoprotein E* (*ApoE*)^−/−^ mouse plaques with high susceptibility to IPH. Subsequently, SDT inhibited iron‐overload‐induced foam‐cell formation and pro‐inflammatory cytokines secretion in vitro. Moreover, SDT reduced levels of the labile iron pool and *ferritin* expression via the reactive oxygen species (ROS)‐nuclear factor erythroid 2‐related factor 2 (Nrf2)‐FPN1 pathway. SDT exerted therapeutic effects on hemorrhagic plaques and reduced iron retention via the ROS‐Nrf2‐FPN1 pathway in macrophages, thereby suggesting that it is a potential translational strategy for patients with advanced atherosclerosis in clinical practice.

## INTRODUCTION

Currently, an existing challenge to antithrombotic treatment for atherosclerotic vascular diseases is the higher frequencies of intraplaque hemorrhage (IPH).^1^, ^2^ IPH plays a major role in the aggressive progression of atherosclerotic plaque, consequently leading to acute cardiovascular events.^3^

The potent atherogenic stimulus caused by IPH is attributed to the deposition of erythrocyte lysis products,^4^ which cause not only cholesterol deposits but also iron retention in plaques.^3^ Iron‐mediated oxidative injury potentiates human atherogenesis^5^ and increases the risk of plaque destabilization.^6^ Additionally, free iron and iron‐binding proteins derived from hemoglobin‐degradation products increase the labile iron pool (LIP) in phagocytes of the plaque, especially in macrophages. The labile nature of LIP is revealed by its capacity to promote reactive oxygen species (ROS) generation via Fenton and Haber–Weiss reactions.^7^ Ferroportin 1 (FPN1), an essential iron exporter identified in mammals, is regulated by hepcidin‐mediated internalization and degradation.^8^ FPN1 levels insufficient to maintain iron homeostasis in cells with iron overload results in excessive cyclic production of ROS, leading to oxidative‐stress injury. Therefore, FPN1 upregulation or FPN1‐activity restoration might represent a novel approach to treating iron‐loaded hemorrhagic plaque.

Established clinical anti‐atherosclerotic strategies intended to control risk factors (e.g., antihypertensive medications and statins) have not manifested significant benefits in treating hemorrhagic plaque.^9^, ^10^ Iron‐deprivation treatments (e.g., iron chelators and hepcidin inhibitors) reportedly exhibit anti‐atherosclerotic effects^11^, ^12^ but can cause systemic imbalances in iron metabolism, leading to adverse effects, such as infection^13^ and audiovisual‐perception impairment.^14^ Therefore, novel tissue‐specific and efficient therapy is in great demand.

Sonodynamic therapy (SDT) has been proposed as a non‐invasive approach to treating tumor and atherosclerosis.^15^, ^16^, ^17^ SDT generates different concentrations of ROS based on the synergistic effects of sonosensitizers and ultrasound. The sonosensitizers used for SDT are mainly porphyrin derivatives. Among these, sinoporphyrin sodium (DVDMS) is a novel sonosensitizer isolated from Photofrin which has been approved by the Food and Drug Administration as a sensitizer. DVDMS has higher chemical purity, better water solubility, stronger sonoactivity, and less skin sensitivity.^18^, ^19^ Therefore, DVDMS has priority in SDT application. Moreover, compared with tumor treatment, lower intensity of ultrasound is applied for atherosclerosis treatment.^20^

Our previous studies showed that SDT promotes atherosclerotic plaque stability and regression by targeting macrophages.^15^, ^16^, ^17^ Macrophages play a central role in iron metabolism^21^; therefore, in the present study, we used animal models and in vitro experiments to clarify whether SDT rescued complicated plaques with IPH and modulated plaque‐specific iron metabolism, as well as address the underlying mechanisms.

## MATERIALS AND METHODS

A detailed description of the materials and methods is available in the Online Supplement.

### Animals

**FIGURE 1:**
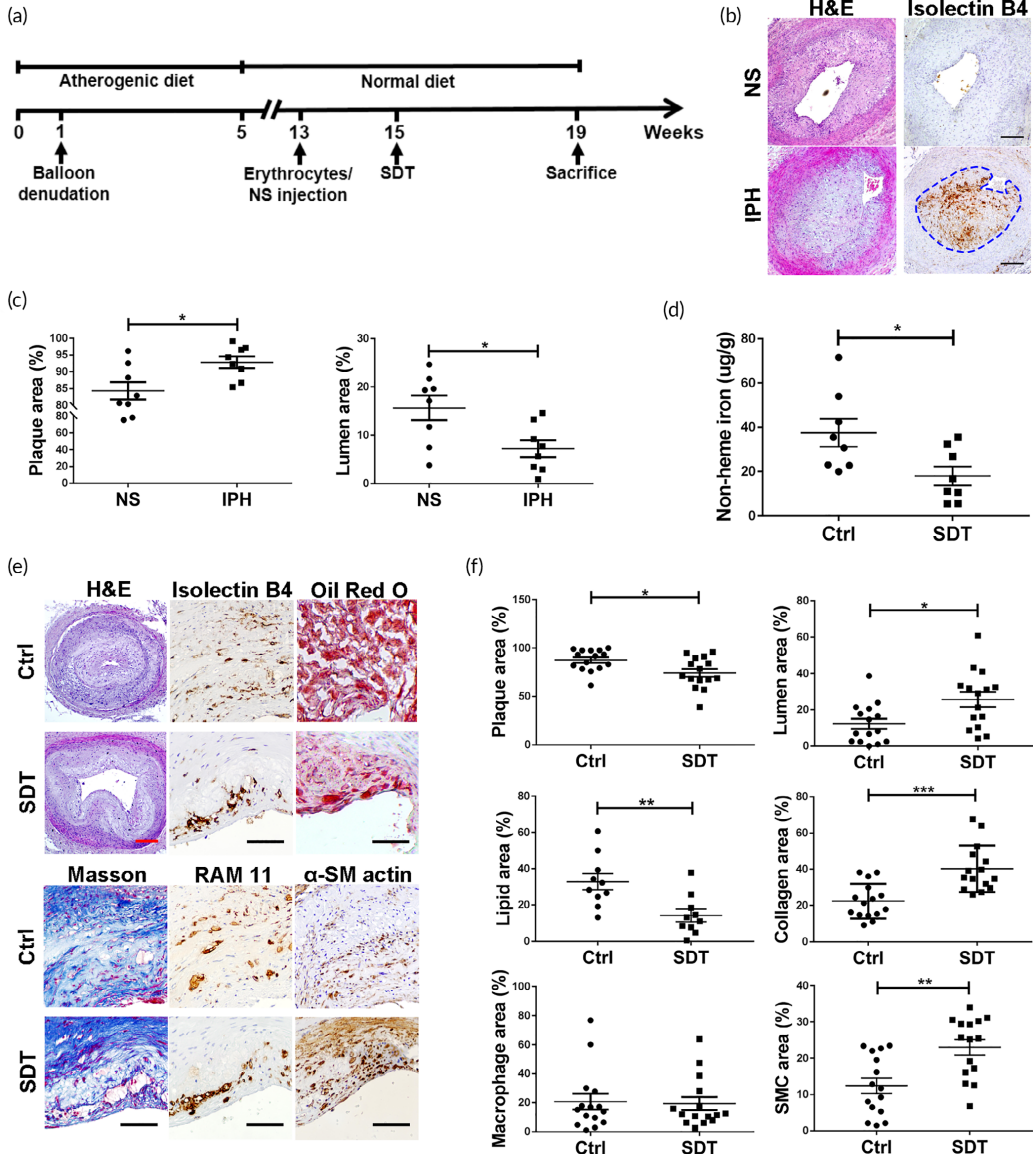
Sonodynamic therapy (SDT) reduces iron retention and exerts anti‐atherosclerotic effects on rabbit hemorrhagic plaque. (a) Schematic diagram describing establishment of the rabbit model and SDT treatment. (b) Immunohistochemistry (IHC) staining of rabbit plaques shows that glycophorin A (stained with isolectin B4) in plaque of the intraplaque hemorrhage (IPH) group; dotted lines indicate the hemorrhagic area in the plaques of the IPH group. Hematoxylin and eosin (H&E) staining and (c) relative quantification shows that IPH promoted plaque progression compared with normal saline (NS) group. (*n* = 8/group). Scale bar, 250 μm. (d) SDT reduced nonheme iron levels of rabbit hemorrhagic plaques (*n* = 8/group). (e,f) Histopathological staining and relative quantification show that SDT induced size and composition changes in rabbit hemorrhagic plaques (H&E staining, Masson staining, IHC staining [RAM11: macrophage, α‐SM actin: smooth muscle cell]: *n* = 15/group; oil red O staining: *n* = 10/group). Red scale bar, 250 μm; black scale bar, 50 μm. *p < 0.05, **p < 0.01, *** *p* < 0.001

Animal experiment protocols were approved by the Ethics Committee of Harbin Medical University. All applicable institutional and national guidelines for the care and use of animals were followed. Male New Zealand rabbits (age: 3–4 months; 2.5–3.0 kg) were purchased from Solarbio Bioscience & Technology Co., Ltd. (Shanghai, China). Male a*polipoprotein E (ApoE*)^−/−^ mice (age: 6 weeks) and 12–14‐week‐old C57BL/6 mice were purchased from Qingzilan Technology Co., Ltd. (Nanjing, China). All efforts were made to minimize animal suffering and reduce the number of animals used. Figure 1(a) and Figure S1(a) depict the flowchart describing the establishment and interventions of the rabbit model and mouse model, respectively. Expanded methods are available in the Online Supplement.

### Isolation of murine peritoneal macrophages and iron‐loaded macrophage formation

C57BL/6 mice were intraperitoneally injected with 3% thioglycolate before isolating macrophages from the peritoneal cavity. After the mice were euthanized, 5 ml of ice‐cold phosphate‐buffered saline (PBS) containing 3% fetal bovine serum (FBS) was injected for peritoneal cavity perfusion. Peritoneal cells were harvested and plated on cell‐culture dishes at appropriate concentrations. After 2 h, the medium was replaced with Roswell Park Memorial Institute (RPMI)‐1,640 containing 12% FBS for adherent cell culture. Macrophages were incubated with 100 μM ferric ammonium citrate (FAC) for 12 h to allow iron‐loaded macrophage (ILM) formation. Expanded methods are available in the Online Supplement.

### SDT treatment

The ultrasound device was manufactured by Harbin Institute of Technology (Harbin, China). The ultrasonic transducer parameters were as follows: diameter, 35 mm; and resonance frequency, 1.0 MHz. DVDMS was used as the sonosensitizer for SDT.

The animals were kept away from light during and after SDT treatment for 24 h. At 4 h after intravenous DVDMS (4 mg/kg) administration, anesthetized animals were subjected to ultrasound for 15 min with an ultrasonic intensity of 1.5 W/cm^2^ for rabbits and 0.4 W/cm^2^ for mice, as previously described.^17^, ^22^ Mice in the hepcidin + SDT group were intraperitoneally administered 25 μg human hepcidin‐25 dissolved in 100 μl PBS 1 h before SDT treatment, whereas mice in the hepcidin group received only hepcidin‐25. The ultrasound was applied as previously described.^15^ In brief, for rabbits, the ultrasonic transducer was placed on the marked femoral artery through a degassed water column, whereas for mice, the ultrasonic transducer was placed under the neck through a degassed water column.

For the in vitro study, cells were plated on 35‐mm Petri dishes that were placed on a degassed water bath 30 cm away from the ultrasonic transducer. Based on the optimized SDT parameters (Figure S2(a)–(c)), ILMs were incubated with 0.2 μM DVDMS for 4 h, followed by irradiation with an ultrasound intensity of 0.2 W/cm^2^ for 5 min.

### Statistical analysis

All data are presented as the mean ± standard error of the mean. The normality test was performed to determine whether the data were normally distributed, and in cases where data were distributed normally, data were analyzed using a Student's *t* test, one‐way analysis of variance (ANOVA), followed by Tukey's multiple comparison test or Dunnett's multiple comparison test, or two‐way ANOVA, followed by Sidak's multiple comparison test, as appropriate. For data that were not normally distributed, a Kruskal–Wallis test, followed by Dunn's multiple comparison test, was used. Statistical analyses were performed using GraphPad Prism 7.0 (GraphPad Software, San Diego, CA, USA), and *p* < 0.05 was considered statistically significant.

## RESULTS

### SDT reduces iron retention and exerts anti‐atherosclerotic effects in rabbit hemorrhagic plaque

The rabbit IPH model was established as shown in Figure 1(a) and (b), and the hemorrhagic area could be observed in the plaques of the IPH group. Compared with the normal saline group, IPH enlarged the plaque size (Figure 1(c)). To evaluate the effects of SDT on hemorrhagic plaque, rabbits with femoral hemorrhagic plaques were randomly assigned to Ctrl and SDT groups. We observed a significant decrease in nonheme iron content in rabbit hemorrhagic plaques after SDT treatment (Figure 1(d)). Moreover, compared with that in the Ctrl group, plaque size in the SDT group decreased, and the artery lumen was enlarged by 13%. Additionally, the oil red O‐positive area was significantly reduced by 18% in SDT‐treated plaques, whereas the collagen content increased by 18%. No changes were found in macrophage content, whereas the proportion of smooth muscle cells increased following SDT treatment (Figure 1(e) and (f)). These data demonstrated that SDT reduced iron retention and positively altered the composition of rabbit hemorrhagic plaque.

### SDT modifies iron metabolism in plaques of ApoE ^−/−^ mice

**FIGURE 2:**
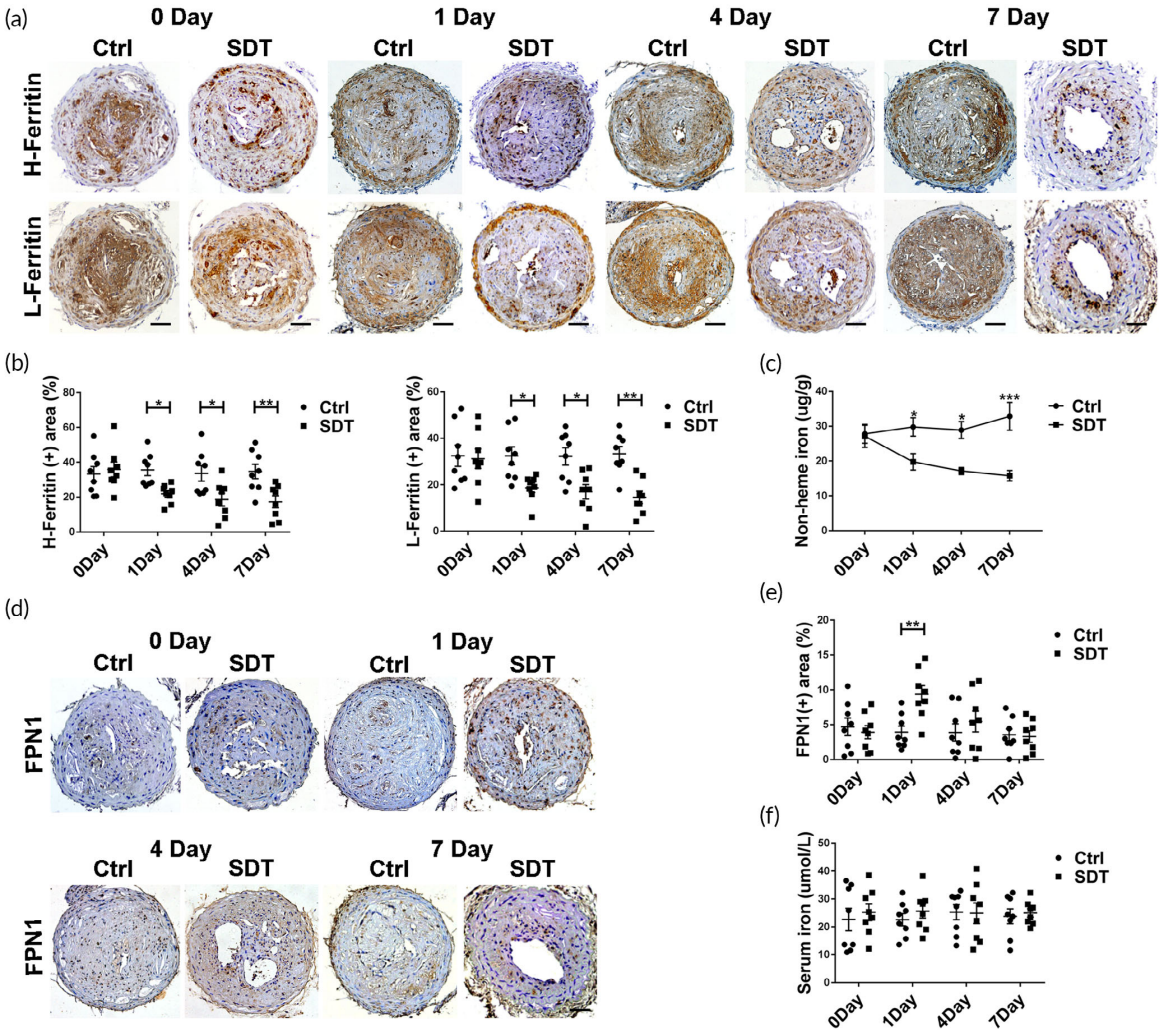
Sonodynamic therapy (SDT) affects iron metabolism in *ApoE* ^*−/−*^ mouse plaques with high susceptibility of intraplaque hemorrhage (IPH). (a,b) Immunohistochemistry (IHC) staining and relative quantification show dynamic changes in H‐ and L‐ferritin levels in mouse plaques after SDT (*n* = 8/group). Scale bar, 25 μm. (c) Dynamic alteration of nonheme iron levels in mouse plaques after SDT (*n* = 4/group). (d,e) IHC staining and relative quantification show that SDT promoted *Fpn1* expression on day 1 (*n* = 8/group). Scale bar, 25 μm. (f) SDT had no effect on serum iron levels in mice (*n* = 8/group). *p < 0.05, **p < 0.01, ***p < 0.001

Consistently, SDT treatment of plaques with high susceptibility to IPH in *ApoE* ^−/−^ mice exerted anti‐atherosclerotic effects (Figure S3). We then evaluated changes in levels of proteins associated with iron metabolism (ferritin, FPN1, and hepcidin) and nonheme iron in plaques of *ApoE* ^*−/−*^ mice. Ferritin, including H‐ and L‐ferritin, reflects iron‐storage levels.^23^ We found that both H‐ and L‐ferritin levels gradually decreased over time in SDT‐treated mouse plaques. At Day 7 after SDT treatment, H‐ and L‐ferritin levels were reduced by 17% and 19% in plaques, respectively (Figure 2(a) and (b)), and nonheme iron content in plaques decreased in a time‐dependent manner following SDT treatment (Figure 2(c)), although no obvious change in the media H‐ and L‐ferritin levels of mouse arteries (Figure S4) and serum iron levels (Figure 2(f)) were observed. Furthermore, FPN1 is internalized and degraded by hepcidin and represents the main regulating factor of iron efflux.^8^ As expected, *Fpn1* expression in plaques was significantly enhanced on Day 1 after SDT (Figure 2(d) and (e)); however, neither local nor systemic hepcidin levels were significantly altered at 1 week after SDT treatment (Figure S1(b)–(d)), suggesting that FPN1 rather than hepcidin is the key protein contributing to reduction in iron retention in plaques following SDT treatment.

### SDT reduces iron retention in the plaque of ApoE ^−/−^ mice by inducing Fpn1 expression

**FIGURE 3:**
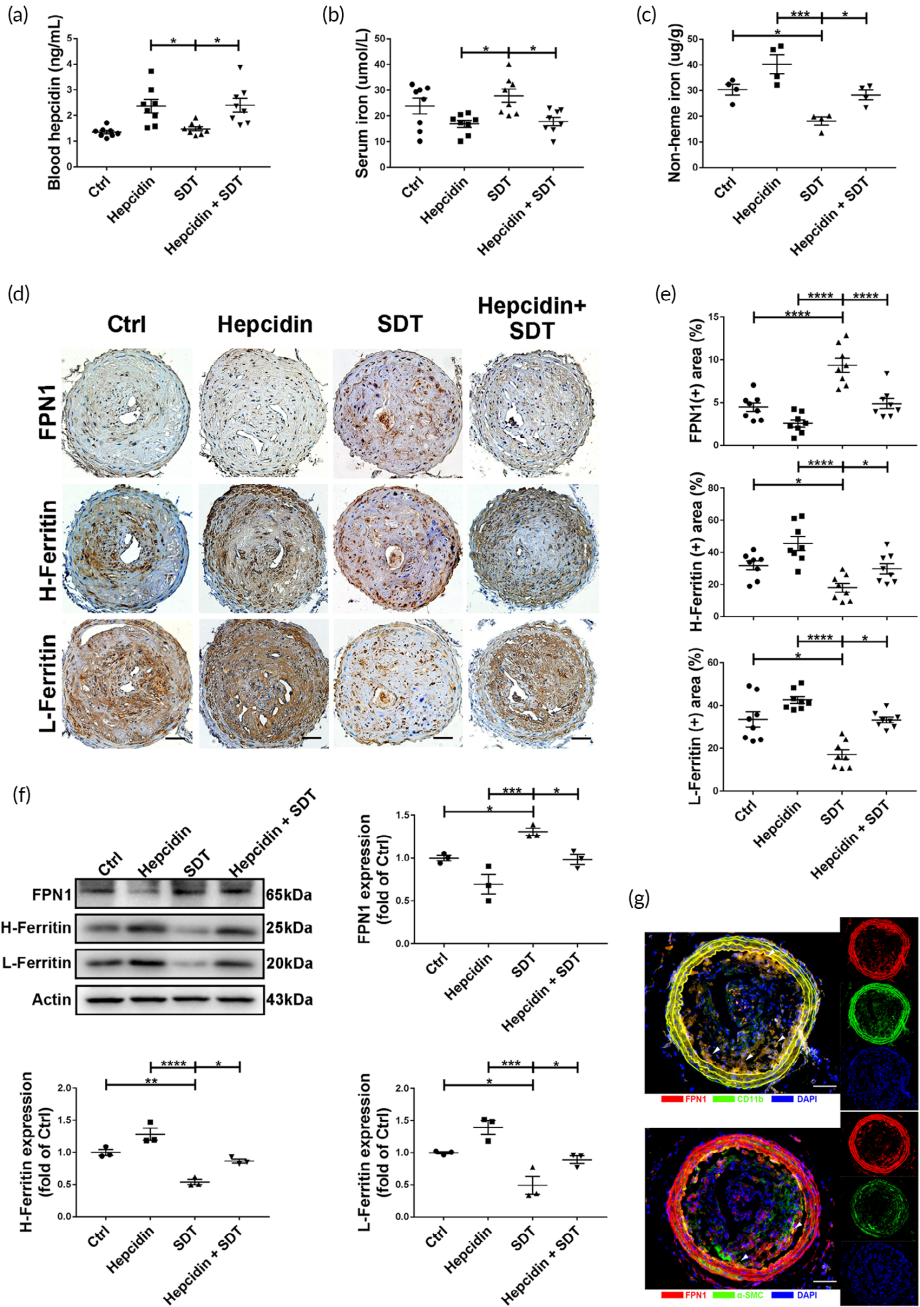
Sonodynamic therapy (SDT) reduces iron retention of plaques by inducing Fpn1 expression in *ApoE* ^*−/−*^ mice. (a) Serum hepcidin and (b) iron levels in mice from different groups (*n* = 8/group). (c) SDT decreased nonheme iron levels in mouse plaques, which was inhibited by hepcidin administration (*n* = 4/group). (d,e) Immunohistochemistry (IHC) staining and relative quantification show that SDT increased FPN1 levels and decreased H‐ and L‐ferritin levels in mouse plaques, which was reversed by hepcidin administration (*n* = 8/group). Scale bar, 25 μm. (f) Western blot analysis and relative quantification show that SDT increased FPN1 levels and decreased H‐ and L‐Ferritin levels in mice plaques, which was reversed by hepcidin administration (*n* = 3/group). (g) Immunofluorescence shows that FPN1 was mainly expressed in macrophages in mouse plaque. (Top) Blue: nucleus; red: FPN1; and green: macrophages. (Below) Blue: nucleus; red: FPN1; and green: smooth muscle cells. Arrowheads indicate positive double‐staining areas. Scale bar, 25 μm. *p < 0.05, **p < 0.01, ***p < 0.001

To address the mechanism of how SDT reduces iron retention in plaque, *ApoE* ^*−/−*^ mice (the hepcidin and hepcidin + SDT groups) were given intraperitoneal injections of hepcidin to induce systemic FPN1 degradation. We observed increases in serum hepcidin levels and decreases in serum iron levels in hepcidin‐treated mice (Figure 3(a) and (b)), confirming that hepcidin promoted systemic degradation of FPN1. At Day 1 after SDT treatment, nonheme iron content in plaques decreased by >10% compared with that in the Ctrl group, which was reversed by hepcidin treatment (Figure 3(c)). Additionally, *Fpn1* expression in SDT‐treated plaque increased, whereas H‐ and L‐ferritin levels decreased by 13% and 11%, respectively (Figure 3(d) and (e)), with SDT‐induced increases in FPN1 levels and decreases in H‐ and L‐ferritin levels reversed by hepcidin treatment according to western blot analysis (Figure 3(f)). Furthermore, immunofluorescence analysis to identify the major cell type expressing *Fpn1* in plaques revealed FPN1 co‐localization with macrophages (Figure 3(g)), suggesting that SDT influenced *Fpn1* expression in macrophages as the dominant target cell.

### SDT reduces intracellular iron content of cultured ILMs by inducing Fpn1 expression

**FIGURE 4:**
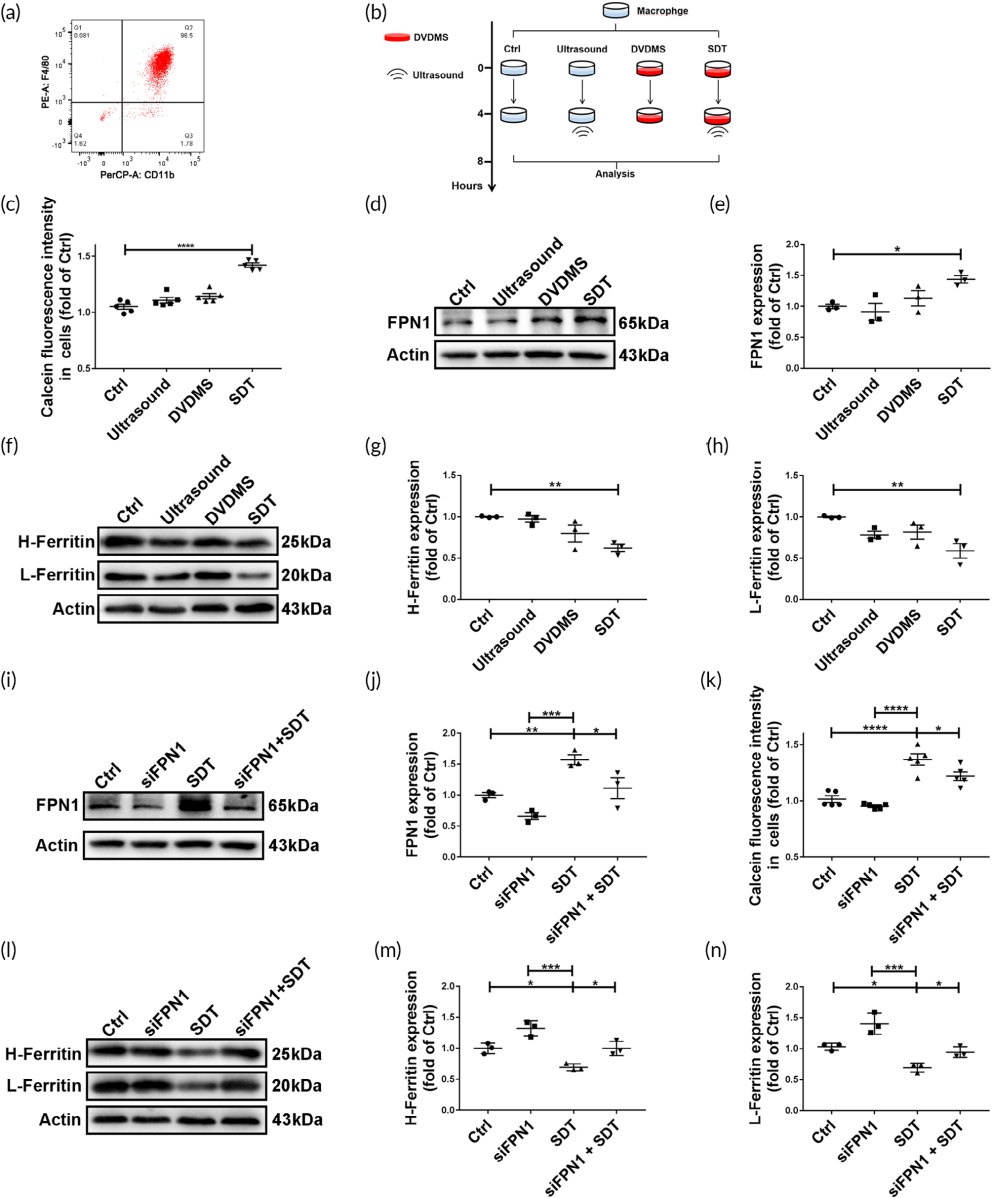
Sonodynamic therapy (SDT) reduces intracellular iron in macrophages by inducing *Fpn1* expression. (a) Identification of murine peritoneal macrophages by flow cytometry using the macrophage markers CD11b and F4/80. (b) Schematic diagram illustrating the process of SDT treatment in vitro. (c) Calcein fluorescence intensity shows that SDT rather than ultrasound or sonosensitizer (DVDMS) decreased labile iron pool (LIP) levels in iron‐loaded macrophages (ILMs) (*n* = 5/group). Intracellular calcein fluorescence is inversely related to free iron levels. (d,e) Western blot analysis and relative quantification show that SDT increased FPN1 levels in ILMs (*n* = 3/group). (f–h) Western blot analysis and relative quantification show that SDT decreased H‐ and L‐ferritin levels in ILMs (*n* = 3/group). (i,j) Western blot analysis and relative quantification show that *Fpn1* siRNA (siFPN1) inhibited SDT‐induced FPN1 levels in ILMs (*n* = 3/group). (k) Calcein fluorescence intensity shows that siFPN1 inhibited SDT‐induced reductions in the LIP in ILMs (*n* = 5/group). (l–n) Western blot analysis and relative quantification show that siFPN1 inhibited decreases in H‐ and L‐ferritin levels induced by SDT in ILMs (*n* = 3/group). *p < 0.05, **p < 0.01, ***p < 0.001, ****p < 0.0001

Murine peritoneal macrophages were identified by flow cytometry (Figure 4(a)). To investigate whether SDT directly affects iron metabolism in macrophages, ILMs were cultured and treated using optimized SDT parameters (see the schematic diagram in Figure 4(b)), and Calcein‐AM was used to determine intracellular LIP in ILMs 24 h after SDT treatment. As shown in Figure 4(c), SDT rather than ultrasound or DVDMS alone significantly reduced the LIP in ILMs. Additionally, we consistently observed enhanced expression of *Fpn1* 4 h after SDT treatment (Figure 4(d) and (e); Figure S2(d), as well as reductions in H‐ and L‐ferritin levels following SDT (Figure 4(f)–(h)). To investigate whether the enhanced *Fpn1* expression accounted for the reduced iron retention caused by SDT, *Fpn1* was knocked down with siRNA in macrophages before FAC incubation (Figure 4(i)–(k)). As expected, *Fpn1* siRNA reversed the iron‐reduction effect of SDT (Figure 4(l)–(n)).

### SDT inhibits macrophage‐derived foam cell formation and pro‐inflammatory cytokine secretion

**FIGURE 5:**
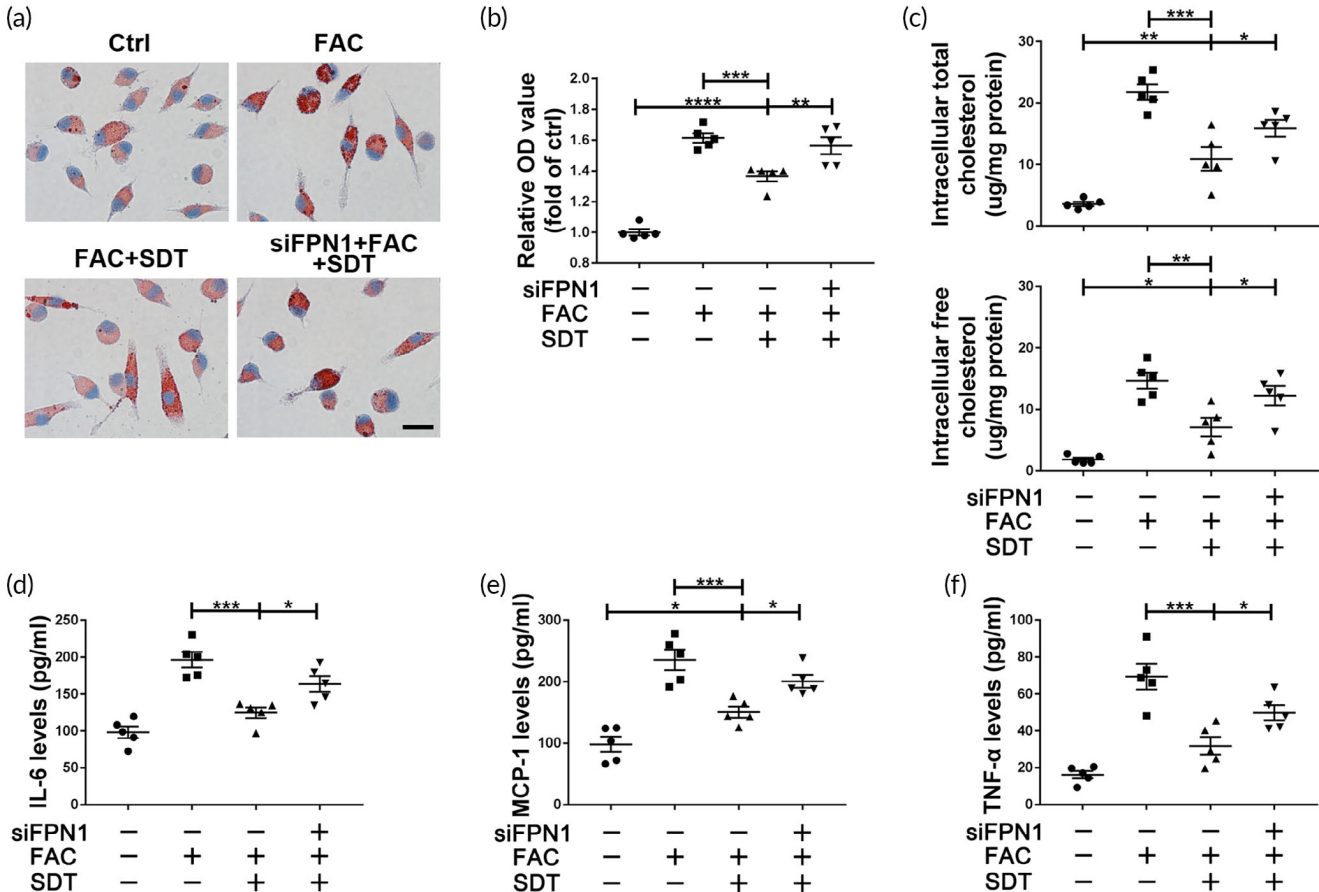
Sonodynamic therapy (SDT) inhibits formation of macrophage‐derived foam cells and reduces pro‐inflammatory cytokine secretion. (a,b) Oil red O staining and subsequent isopropanol‐extraction optical density scan show that SDT inhibited ferric ammonium citrate (FAC)‐induced lipid accumulation in macrophages, which was reversed by *Fpn1* siRNA (siFPN1) (*n* = 5/group). (c) SDT decreased FAC‐induced increases in intracellular total and free cholesterol levels, with this activity inhibited by siFPN1 (*n* = 5/group). (d–f) SDT decreased FAC‐induced elevations in pro‐inflammatory cytokine MCP‐1, TNF‐α, and IL‐6 levels in macrophage culture supernatants, with this activity inhibited by siFPN1 (*n* = 5/group). *p < 0.05, **p < 0.01, ***p < 0.001, ****p < 0.0001

Intracellular iron overload is a strong stimulus for foam cell transformation and pro‐inflammatory cytokine secretion by macrophages. In vivo, SDT reduced lipid and pro‐inflammatory cytokine levels (IL‐6, MCP‐1, and TNF‐α) in plaque (Figure 1(e) and (f); and Figure S5(a) and (b)). In vitro, SDT induced intracellular iron depletion in ILMs, thereby inhibiting foam cell transformation and reducing intracellular total and free cholesterol (Figure 5(a)–(c)), as well as inhibiting pro‐inflammatory cytokines (IL‐6, MCP‐1, and TNF‐α) secretion (Figure 5(d)–(f)).

### SDT attenuates iron overload in ILMs by activating ROS‐Nrf2‐FPN1 signaling

**FIGURE 6:**
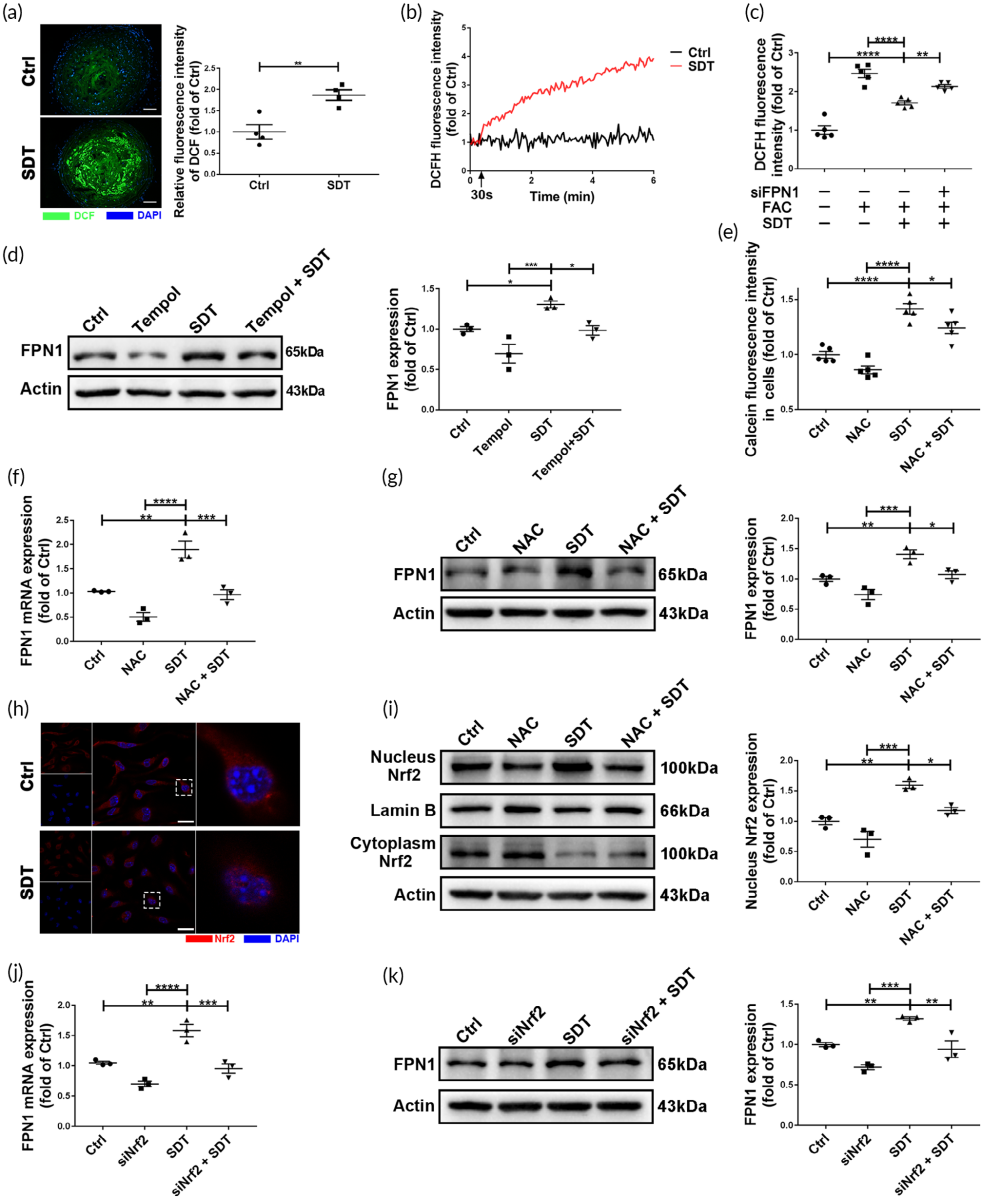
Sonodynamic therapy (SDT) alleviates iron overload in iron‐loaded macrophages (ILMs) by activating reactive oxygen species (ROS)‐Nrf2‐FPN1 signaling. (a) DCF fluorescence shows that SDT induced ROS generation in mouse plaques immediately (*n* = 4/group). Blue: nucleus; green: ROS. Scale bar, 25 μm. (b) Real‐time monitoring shows that SDT promoted ROS generation in ILMs during ultrasonic irradiation. (c) DCF fluorescence shows that SDT decreased ferric ammonium citrate (FAC)‐induced increases in intracellular ROS levels at 24 h, with this activity inhibited by *Fpn1* siRNA (siFPN1) (*n* = 5/group). (d) Western blot analysis and relative quantification show that ROS scavenger 4‐hydroxy‐TEMPO (Tempol) inhibited SDT‐induced increases in FPN1 levels in mouse plaques (*n* = 3/group). (e) Calcein fluorescence sshows that ROS scavenger N‐acetylcysteine (NAC) inhibited SDT‐induced reductions in the intracellular labile iron pool (LIP) in ILMs (*n* = 5/group). (f) Real‐time PCR analysis shows that SDT increased *Fpn1* mRNA levels in ILMs, with this activity inhibited by NAC (*n* = 3/group). *Fpn1* mRNA levels were quantified relative to *Gapdh* mRNA. (g) Western blot analysis and relative quantification show that NAC inhibited SDT‐induced increases in FPN1 levels in ILMs (*n* = 3/group). (h) SDT‐induced nuclear accumulation of Nrf2 observed by laser scanning confocal microscopy. Blue: nucleus; red: Nrf2. Scale bar, 20 μm. (i) Western blot analysis and relative quantification show increases in Nrf2 protein in the nucleus of ILMs after SDT, with this activity reversed by NAC (*n* = 3/group). (j) Real‐time PCR analysis shows that *Nrf2* siRNA (siNrf2) inhibited increases in SDT‐induced *Fpn1* mRNA levels in ILMs (*n* = 3/group). *Fpn1* mRNA levels were quantified relative to *Gapdh* mRNA. (k) Western blot analysis and relative quantification show that siNrf2 inhibited SDT‐induced increases in FPN1 levels in ILMs (*n* = 3/group). *p < 0.05, **p < 0.01, ***p < 0.001, ****p < 0.0001

SDT exerts its biological effects through ROS production.^24^ As shown in Figure 6(a), the production of ROS in plaque increased instantly after SDT treatment. However, at Day 7 after SDT treatment, ROS levels within plaques in the SDT group was significantly lower than that in the Ctrl group, which was reversed by hepcidin treatment (Figure S6). To confirm ROS generation in ILMs upon SDT treatment, intracellular ROS levels were monitored during ultrasonic irradiation. Compared with those in Ctrl cells, intracellular ROS levels were markedly elevated during SDT ultrasonic irradiation (Figure 6(b)) but decreased to levels lower than those in untreated ILMs after 24 h (Figure 6(c)). These results suggest an acute temporary elevation of ROS levels during SDT treatment in the absence of enduring ROS‐induced cell or tissue injury.

To clarify whether SDT‐induced elevations in Fpn1 expression is related to activation of ROS‐specific signaling, we performed 4‐hydroxy‐TEMPO (Tempol) or N‐acetylcysteine (NAC) pretreatment to scavenge ROS in vivo or vitro. The results showed that Tempol pretreatment significantly inhibited SDT‐induced Fpn1 expression in vivo (Figure 6(d)). Meanwhile, NAC pretreatment inhibited SDT‐induced intracellular LIP reduction and Fpn1 expression (Figure 6(e) and (f)). Because Fpn1 mRNA levels increased after SDT (Figure 6(f)), we performed bioinformatics analysis to identify potential transcription factors regulating Fpn1 (Slc40A1) transcription. The results showed that only nuclear factor erythroid 2‐related factor 2 (Nrf2 [Nfe2l2]) significantly correlated with Slc40A1 transcription (cor = 0.42; *p* < 2.2 × 10^–16^). The genomic location of the Slc40A1 locus and the transcription factor binding site of Nfe2l2 in the Slc40A1 promoter region are shown in Figure S7(a), and the Nfe2l2‐binding motif is shown in Figure S7(b). Nrf2 is an ROS‐activated transcription factor that interacts with the antioxidant response element (ARE) and plays a protective role during the anti‐oxidation processes.^25^ In the present study, SDT increased nuclear accumulation of Nrf2 in ILMs, which was effectively reversed by NAC pretreatment (Figure 6(h) and (i)). Moreover, the SDT‐induced elevations in mRNA and protein levels of FPN1 were reversed by Nrf2 siRNA (Figure 6(j) and (k)), suggesting that SDT alleviated ILM‐specific iron overload by activating ROS‐Nrf2‐FPN1 signaling.

## DISCUSSION

IPH is a major diagnostic occurrence in vulnerable atherosclerotic plaques, which in turn promotes lesion development and instability. To date, hemorrhagic plaque treatment remains a major challenge. In the present study, we attempted to promote the future clinical application of SDT by investigating the therapeutic efficacy of SDT on hemorrhagic plaque. Our in vivo and in vitro studies revealed that SDT: (a) inhibited iron‐overload‐induced foam cell formation and pro‐inflammatory cytokine secretion, thereby attenuating the progression of lesions with IPH; and (b) alleviated iron retention by stimulating FPN1 expression both in vivo and in vitro through activation of ROS‐Nrf2‐FPN1 signaling.

To investigate the effect of SDT on hemorrhagic plaque, a rabbit IPH model was used. Although rabbit abdominal aortic IPH model was reported by delivering autologous erythrocytes into the plaques,^26^ given the negative effect of intestinal gas on ultrasonic irradiation, this model is not suitable for SDT treatment. Therefore, in this study, we established a rabbit femoral artery IPH model. Glycophorin A in erythrocyte membranes scattered within the plaque confirmed successful model establishment. With the rabbit IPH model, we revealed that SDT exerts anti‐atherosclerotic effects on hemorrhagic plaques, which is accompanied by the intraplaque iron retention reduction.

Iron‐mediated oxidative stress caused by IPH is a potent potentiator of plaque progression.^5^ Our previous study indicated that SDT inhibits early stage atherosclerosis progression and upregulates heme oxygenase‐1 (HO‐1) levels.^17^ HO‐1, which is regulated by Nrf2, is a protective factor against atherogenesis but can still catabolize heme into free ferric ions.^25^ However, in the murine model, we observed significant reduction of iron retention in advanced hemorrhagic plaque but not in the media after SDT, which was reversed by hepcidin. These results suggest that the predominant impact of SDT was due to upregulated Fpn1 expression in cells within the plaque, especially in the abundant macrophages recruited in response to IPH, which are the target cells of SDT. Moreover, this process is not accompanied by serum iron or serum hepcidin levels changing, suggesting that SDT modulated iron metabolism in a more tissue‐specific manner rather than systemically, which will definitely decrease off‐target effects and systemic side effects.

**FIGURE 7:**
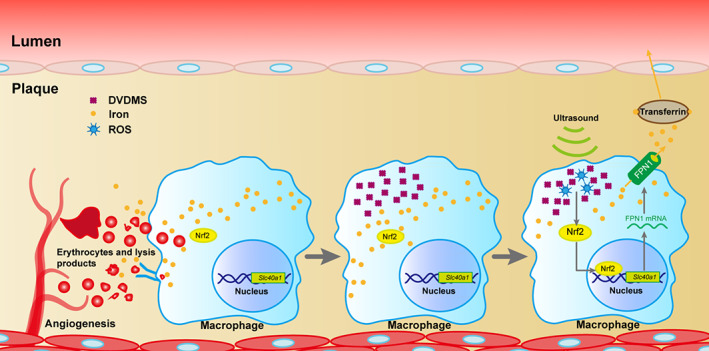
A proposed theoretical model of sonodynamic therapy (SDT) stabilization of plaque with intraplaque hemorrhage (IPH). DVDMS accumulates in iron‐loaded macrophages in hemorrhagic plaque. During ultrasonic irradiation, reactive oxygen species (ROS)‐Nrf2‐FPN1 signaling is active due to increases in intracellular ROS levels. This process promotes iron efflux in macrophages and plaque, resulting in inhibition of foam‐cell transformation, as well as alleviation of inflammatory responses

Mechanistically, SDT reduced iron overload via ROS‐Nrf2‐FPN1 signaling. ROS levels are temporarily increased during SDT, which can deplete cytosolic thiol and thus induce Kelch‐like ECH‐associated protein 1/Nrf2 heterodimer dissociation and Nrf2 translocation to the nucleus to initiate *Fpn1* expression via interaction with the ARE.^27^ At 24 h after SDT, we observed lower intracellular ROS levels; therefore, we speculate that SDT‐mediated increases in FPN1 levels via ROS‐Nrf2‐FPN1 signaling relieved intracellular iron overload, resulting in suppression of ROS production induced by iron overload. Moreover, *Fpn1* siRNA eliminated the suppressive effects of SDT on foam cell formation and pro‐inflammatory cytokines secretion, indicating that reduced iron retention represented an upstream alteration induced by SDT. Consistently, these results support previous reports indicating that iron‐induced oxidative stress promotes foam cell transformation^11^ and increases pro‐inflammatory cytokine secretion.^28^ In addition, it is also suggested that the temporary explosive increase of ROS may activate the antioxidant stress pathway, thus inhibiting the oxidative stress caused by iron overload in macrophages.

Depending on whether SDT increases macrophage apoptosis, it can be divided into the following types: low‐intensity SDT^15^ and very low‐intensity SDT, also known as nonlethal SDT (NL‐SDT).^17^ Although low‐intensity ultrasound based on our previous study was used to treat rabbit hemorrhagic plaque,^22^ no significant increase in the rate of macrophage apoptosis was observed in rabbit plaques following SDT treatment in this study (Figure S8(a) and (b)). After reviewing the rabbit femoral artery ultrasound images, we found that the subcutaneous tissue above the artery in the rabbit model of this study was thicker than that observed in the early plaque model (Figure S8(c) and (d)). The thickened subcutaneous tissue, which was due to the repeated surgical procedures in this study, could aggravate the attenuation of ultrasonic intensity. Therefore, NL‐SDT parameters were applied to murine vulnerable‐plaque model prone to IPH according to previous study.^17^ Furthermore, in vitro study, the ultrasonic intensity of SDT (0.2–0.3 W/cm^2^) resulting in elevated FPN1 levels was lower than that previously used to enhance cell apoptosis (0.5 W/cm^2^).^16^ Interestingly, SDT with 0.5 W/cm^2^ ultrasonic intensity did not increase FPN1 levels. These results indicated that reduced iron retention in hemorrhagic plaque and the anti‐atherosclerotic effects were manifested by NL‐SDT and different signaling pathways were activated in the presence of variable ultrasonic intensity. In addition, this low‐energy SDT may avoid tissue damage caused by excessive ultrasound intensity, thus promoting its future clinical application.

A previous study showed that atherosclerotic carotid plaques obtained from men had a higher prevalence of IPH compared with those obtained from women,^29^ and physiological iron loss before menopause in women has been proposed to be a cardiovascular protective factor.^30^ However, the incidence of carotid IPH and plaque morphology in women with increasing age postmenopause becomes closer to that of men.^31^, ^32^ In this study, the animal model was established using only males, whereas the effect of SDT on hemorrhagic plaque in female animals was not verified. This is a limitation of this study. The effect of SDT on atherosclerotic plaque of individuals with different gender and different physiological periods needs to be further explored and verified in clinical research.

Our recently published clinical study (NCT03457662) showed that SDT rapidly reduced plaque inflammation and improved walking performance among patients with symptomatic peripheral artery disease.^33^ This clinical study mainly focused on the treatment of patients with hypoechoic femoral artery plaque that usually tended to be lipid plaque. Although the clinical study is encouraging to promote the clinical practice of SDT on atherosclerosis treatment, the safety and efficacy of SDT have not yet been confirmed for the treatment of more advanced plaques such as hemorrhagic plaque. The promising results presented herein provided a more convincing rationale to conduct another clinical trial (NCT03871725) for assessing the efficacy and safety of SDT in patients with carotid hemorrhagic plaque, which is a more vulnerable condition. We expect that such studies will promote the rapid development of new approaches to treat vulnerable plaques without systemic side effects.

## CONCLUSION

The hemorrhagic plaque caused by the extensive use of antithrombotic drugs challenges the traditional anti‐atherosclerotic treatments. SDT exerted therapeutic effects on hemorrhagic plaques and reduced iron retention via the ROS‐Nrf2‐FPN1 pathway in macrophages (Figure 7), thereby suggesting that it is a potential translational strategy for patients with hemorrhagic plaque in clinical practice.

## CONFLICT OF INTEREST

The authors declare no conflicts of interest.

## AUTHOR CONTRIBUTIONS

**Bicheng Li**: Conceptualization; formal analysis; investigation; project administration; validation; writing‐original draft; writing‐review and editing. **Jie Gong**: Conceptualization; data curation; formal analysis; investigation; methodology; validation; writing‐original draft. **Siqi Sheng**: Data curation; formal analysis; investigation; project administration; writing‐original draft; writing‐review and editing. **Minqiao Lu**: Formal analysis; investigation; methodology; validation; writing‐original draft. **Shuyuan Guo**: Data curation; formal analysis; investigation; methodology. **Jianting Yao**: Formal analysis; methodology; validation. **Haiyu Zhang**: Data curation; formal analysis; writing‐review and editing. **Xuezhu Zhao**: Formal analysis; investigation; writing‐review and editing. **Zhengyu Cao**: Formal analysis; investigation; methodology. **Xin Sun**: Formal analysis; validation; writing‐review and editing. **Huan Wang**: Formal analysis; validation; writing‐review and editing. **Yang Cao**: Investigation; methodology. **Yongxing Jiang**: Formal analysis; investigation; writing‐review and editing. **Zhen Tian**: Formal analysis; investigation; writing‐review and editing. **Bin Liu**: Methodology; resources; writing‐review and editing. **Hua Zhao**: Methodology; resources. **Zhiguo Zhang**: Methodology; resources. **Hong Jin**: Writing‐review and editing. **Ye Tian**: Conceptualization; funding acquisition; project administration; resources; supervision; writing‐review and editing.

### PEER REVIEW

The peer review history for this article is available at https://publons.com/publon/10.1002/btm2.10193.

## Supporting information


**FIGURE S1 SDT has no effect on hepcidin expression in mice plaque or serum. (A)** Schematic diagram describing establishment of mice model and SDT treatment. **(B, C)** Immunohistochemistry (IHC) staining and relative quantification show SDT did not alter hepcidin expression in mouse plaques (*n* = 8/group). Scale bar, 50 μm. **(D)** SDT had no effect on serum hepcidin levels in mice (*n* = 8/group).
**FIGURE S2.** Optimized parameters of SDT for **ILMs. (A)** The survival rates of iron‐loaded macrophages (ILMs) after sinoporphyrin sodium (DVDMS) incubation at different concentrations (*n* = 3/group). **(B)** Intracellular kinetics of DVDMS fluorescence in ILMs at different time points after incubating with 0.2 μM DVDMS (*n* = 3/group). **(C)** Western blot analysis shows that ferroportin 1 (FPN1) was highly expressed at the 0.2–0.3 W/cm^2^ ultrasound intensity. **(D)** Western blot analysis shows that ILMs had highest expression of FPN1 at 4 h after SDT. ***p < 0.001, ****p < 0.0001.
**FIGURE S3. SDT exerts anti‐atherosclerotic effect on ApoE** ^**−/−**^ **mice plaque with high susceptibility of IPH. (A, B)** Histopathological staining and relative quantification shows that SDT induced size and composition changes in mice haemorrhagic plaques (n = 8/group). Scale bar, 50 μm. *p < 0.05, ***p < 0.001, ****p < 0.0001.
**FIGURE S4. SDT has no effect on Ferritin expression in the media of mouse arteries.** Relative quantification show SDT did not alter H‐ and L‐ferritin levels in media of mouse arteries (n = 8/group).
**FIGURE S5. SDT reduces inflammatory cytokines in rabbit plaque with IPH. (A, B)** IHC staining and relative quantification shows that SDT reduced interleukin‐6 (IL‐6), monocyte chemoattractant protein‐1 (MCP‐1) and tumor necrosis factor‐α (TNF‐α) levels in rabbit hemorrhagic plaque (*n* = 15/group). Scale bar, 50 μm. **p < 0.01, ***p < 0.001, ****p < 0.0001.
**FIGURE S6. SDT reduces Reactive oxygen species (ROS) levels in mouse plaque.** DCF fluorescence shows that the level of ROS within mouse plaque was decreased at day 7 after SDT, which was reversed by hepcidin treatment. Scale bar, 25 μm.
**FIGURE S7**. Bioinformatics analysis results. (A) Slc40a1 localization on chromosome 1, reference sequence and transcription factor binding site. (B) Functional domain prediction of Nfe2l2 (motif).
**FIGURE S8. SDT has no effect on macrophage apoptosis in rabbit plaque with IPH. (A, B)** TUNEL and immunofluorescence double staining and relative quantification show that SDT did not induce macrophage apoptosis in rabbit hemorrhagic plaque. Blue: nucleus; Red: macrophage; green: apoptosis; double red and green staining signifies the macrophages apoptosis. Arrowheads indicate positive double staining areas. (*n* = 8/group). Scale bar, 50 μm. **(C)** Ultrasonography images of rabbit lower limb with plaque model. The ultrasonography images were achieved at 13th week (before secondary surgical operation and erythrocytes injection) and 15th week (2 weeks after secondary surgical operation and erythrocytes injection). The white line indicates the subcutaneous tissue thickness from skin to artery. **(D)** Quantification of the subcutaneous tissue thickness of (C). n = 5 for each group. **p < 0.01.
