# Supplementary material for: Sonodynamic therapy reduces iron retention of hemorrhagic plaque
Source: Bioeng Transl Med. 2020 Oct 31;6(1):e10193. doi: 10.1002/btm2.10193 (PMC7823128; doi:10.1002/btm2.10193)
Supplement: Supplementary file 1 — FIGURE S1 SDT has no effect on hepcidin expression in mice plaque or serum. (A) Schematic diagram describing establishment of mice model and SDT treatment. (B, C) Immunohistochemistry (IHC) staining and relative quantification show SDT did not alter hepcidin expression in mouse plaques (n = 8/group). Scale bar, 50 μm. (D) SDT had no effect on serum hepcidin levels in mice (n = 8/group). FIGURE S2. Optimized parameters of SDT for ILMs. (A) The survival rates of iron‐loaded macrophages (ILMs) after sinoporphyrin sodium (DVDMS) incubation at different concentrations (n = 3/group). (B) Intracellular kinetics of DVDMS fluorescence in ILMs at different time points after incubating with 0.2 μM DVDMS (n = 3/group). (C) Western blot analysis shows that ferroportin 1 (FPN1) was highly expressed at the 0.2–0.3 W/cm2 ultrasound intensity. (D) Western blot analysis shows that ILMs had highest expression of FPN1 at 4 h after SDT. ***p < 0.001, ****p < 0.0001. FIGURE S3. SDT exerts anti‐atherosclerotic effect on ApoE −/− mice plaque with high susceptibility of IPH. (A, B) Histopathological staining and relative quantification shows that SDT induced size and composition changes in mice haemorrhagic plaques (n = 8/group). Scale bar, 50 μm. *p < 0.05, ***p < 0.001, ****p < 0.0001. FIGURE S4. SDT has no effect on Ferritin expression in the media of mouse arteries. Relative quantification show SDT did not alter H‐ and L‐ferritin levels in media of mouse arteries (n = 8/group). FIGURE S5. SDT reduces inflammatory cytokines in rabbit plaque with IPH. (A, B) IHC staining and relative quantification shows that SDT reduced interleukin‐6 (IL‐6), monocyte chemoattractant protein‐1 (MCP‐1) and tumor necrosis factor‐α (TNF‐α) levels in rabbit hemorrhagic plaque (n = 15/group). Scale bar, 50 μm. **p < 0.01, ***p < 0.001, ****p < 0.0001. FIGURE S6. SDT reduces Reactive oxygen species (ROS) levels in mouse plaque. DCF fluorescence shows that the level of ROS within mouse plaque was decreased at da [file BTM2-6-e10193-s001.docx]

**Supplementary Material**

**Sonodynamic therapy reduces iron retention of haemorrhagic plaque**

**Authors:** Bicheng Li, Jie Gong, Siqi Sheng, Minqiao Lu, Shuyuan Guo, Jianting Yao, Haiyu Zhang, Xuezhu Zhao, Zhengyu Cao, Xin Sun, Huan Wang, Yang Cao, Yongxing Jiang, Zhen Tian, Bin Liu, Hua Zhao, Zhiguo Zhang, Hong Jin, Ye Tian

# Supplementary Materials and Methods

## 1.1 Materials

Sinoporphyrin sodium (DVDMS) is the property of Qinglong Hi-tech Co, Ltd, Jiangxi, China and was kindly provided by Professor Qicheng Fang from the Chinese Academy of Medical Sciences, Beijing, China. Thioglycolate was purchased from Sigma-Aldrich, MO, USA. Human hepcidin-25 was synthesized by Bioss Bioscience & Technology Co., Ltd., Beijing, China. Ferric ammonium citrate was purchased from Macklin Co., Ltd, Shanghai, China. Calcein-AM was purchased from Fanbo Biochemicals Co., Ltd., Beijing, China. ReverTra Ace qPCR RT Master Mix and SYBR® Green Real-time PCR Master Mix were purchased from Toyobo Co., Ltd., Japan. Optimum cutting temperature compound was purchased from Sakura Finetechnical Co., Ltd., Tokyo, Japan. DAPI was purchased from Roche Applied Science Inc., Lewes, UK. 4-Hydroxy-TEMPO and N-acetylcysteine were purchased from MedChemExpress Llc., NJ, USA.

The following primary antibodies were used in immunohistochemistry and immunofluorescence analysis: anti-RAM11, (#M0633, Dako, 1:1200), anti-CD68 (#ab955, Abcam, 1:200), anti-FPN1 (#26601-1-AP, Proteintech, 1:200), anti-Ferritin Light Chain (#ab69090, Abcam, 1:200), anti-Ferritin Heavy Chain (#ab65080, Abcam, 1:200), anti-α-Smooth Muscle actin (#A2547, Sigma, 1:400), anti CD11b (#550993, BD Pharmingen, 1:200), GSL Isolectin B4 (B-1205, Vector, 1:100), anti-Hepcidin (#ab30760, Abcam, 1:20), anti-TER-119 (#sc-19592, Santa, 1:200), anti-IL-6 (#bs-6312R, Bioss, 1:400), anti-MCP-1 (#bs-1955R, Bioss, 1:400), anti-TNF-α (#bs-2081R, Bioss, 1:400).

The following primary antibodies were used in Western blot analysis: anti-FPN1 (#125374, Absin, 1:500), anti-Hepcidin (#ab30760, Abcam, 1:50), anti-Ferritin Light Chain (#ab69090, Abcam,1:1000), anti-Ferritin Heavy Chain (#ab65080, Abcam, 1:1000), anti-NRF2 (#16396-1-AP, Proteintech, 1:500), anti-Lamin B1 (#12987-1-AP, Proteintech, 1:1000), anti-β actin (TA-09, Zsbio, 1:1000).

The following antibodies were used in flow cytometry analysis: anti-CD11b (#550993, BD Pharmingen, 1:200), anti-F4/80 (#12-4801-80, eBioscience, 1:100).

**1.2 Rabbit model of intraplaque haemorrhage**

A high-cholesterol diet (1.5% cholesterol, 10% lard and 7.5% yolk powder) was given to the rabbits 1 week before balloon-induced endothelial injury to the right femoral arteries, as previously reported[^1^](#_ENREF_1). Before the surgery, the rabbits were anaesthetized with an intramuscular injection of ketamine (25 mg/kg), xylazine (5 mg/kg) and acepromazine (0.75 mg/kg). Anaesthesia was maintained with 1% isoflurane delivered in oxygen. After the surgery, the rabbits remained on the high-cholesterol diet for 4 more weeks, followed by purified rabbit chow with no cholesterol. Twelve weeks after the surgery, the rabbit femoral arteries were exposed again under anaesthesia, as described, and 25 μL of washed autologous erythrocytes or an equal volume of normal saline (NS) was injected into the plaque by a 1-mL syringe with a 30-gauge needle [^2^](#_ENREF_2). To determine the effects of SDT on plaque with IPH, IPH-model rabbits were randomly assigned to untreated (Ctrl group) and SDT groups at week 15. All the rabbits were sacrificed at the end of week 19.

**1.3 Mouse model with high susceptibility to IPH**

Apolipoprotein E (ApoE)^−/−^ mice were fed on an atherogenic diet (22% fat, 0.15% cholesterol) beginning at 6 weeks of age. Upon reaching 12 weeks of age, the mice underwent ‘tandem stenosis’ surgery to develop plaques prone to developing IPH [^3^](#_ENREF_3). Briefly, after anaesthetization with a ketamine and xylazine mixture administered intraperitoneally, an incision was made in the neck to expose the right carotid artery. Two stenoses with 150-μm outer diameter were made on the common carotid artery. The distance from the stenosis point to carotid bifurcation was 1 mm and 4 mm, respectively. At 7 weeks after surgery, the mice were randomly assigned to different groups, as indicated.

**1.4 Tissue preparation and staining**

Overdose of sodium pentobarbital (100 mg/kg for rabbits, 160 mg/kg for mice) was used to euthanize animals, followed by harvesting of arteries, fixation in 4% paraformaldehyde, and embedding in paraffin. For cryosections, arteries were embedded in optimum cutting-temperature compound and frozen. The paraffin sections were prepared for haematoxylin and eosin, Masson’s trichrome, and immunohistochemistry staining, and cryosections were prepared for oil red O and immunofluorescence staining. Pathological images were analysed by Image-Pro Plus 6.0 (Media Cybernetics, Inc., Silver Spring, MD, USA).

**1.5 Blood sample analysis**

Mouse serum was collected from whole blood, and serum iron and hepcidin levels were determined using an iron assay kit (BioAssay Systems Inc., Hayward, CA, USA) and hepcidin enzyme-linked immunosorbent assay (ELISA) kit (Elabscience Biotechnology Co., Ltd., Wuhan, China) according to manufacturer instructions.

**1.6 Measurement of tissue non-haem iron**

Non-haem iron in arteries was measured using an iron colorimetric assay kit (BioVision Inc., CA, Milpitas, USA), as described previously [^4^](#_ENREF_4). Briefly, harvested carotid arteries were homogenized in iron assay buffer and centrifuged to remove insoluble materials, after which the samples were tested according to manufacturer instructions. Metal devices were avoided during all processes.

## 1.7 Flow cytometric analysis

## Isolated murine peritoneal cells were stained with conjugated antibodies against macrophage markers（CD11b and F4/80）in the dark. Then the cells were determined and analysed with a FACSCalibur flow cytometer (BD Biosciences, Franklin Lakes, NJ, USA).

## 1.8 Measurement of intracellular LIP

Intracellular LIP was measured using Calcein-AM, as described previously [^5^](#_ENREF_5). Intracellular Calcein fluorescence is inversely related to free iron levels [^5^](#_ENREF_5). Macrophages were incubated with 0.5 μM Calcein-AM in RPMI-1640 and analysed using a fluorescence microplate reader (Tecan Group Ltd., Zürich, Switzerland).

## 1.9 Western blot analysis

Total protein from cells or tissues was extracted according to previously reported methods [^6^](#_ENREF_6). Nuclear proteins were extracted using a nuclear and cytoplasmic protein extraction kit (Beyotime Biotechnology Co., Ltd., Shanghai, China) according to manufacturer instructions. Western blot was performed as previously described [^6^](#_ENREF_6).

## 1.10 Real-time polymerase chain reaction (PCR)

Total RNA was isolated using TRIzol reagent (Invitrogen, Carlsbad, CA, USA) and reverse transcribed to cDNA, which was analysed by quantitative SYBR Green PCR. Primer sequences were as follows: *glyceraldehyde 3-phosphate dehydrogenase* (*Gapdh*), Gapdh-F 5′-AGGTCGGTGTGAACGGATTTG-3′ and Gapdh-R 5′-TGTAGACCATGTAGTTGAGGTCA-3′; and *Fpn1*, Slc40a1-F 5′-ACCAAGGCAAGAGATCAAACC-3′ and Slc40a1-R 5′-AGACACTGCAAAGTGCCACAT-3′. Relative mRNA expression was normalized to *Gapdh* mRNA.

## 1.11 Transfection of small-interfering RNA (siRNA)

siRNAs were purchased from GenePharma Co, Ltd. (Shanghai, China). Cells were transiently transfected with *Fpn1* and *Nrf2* siRNA or scrambled siRNA mixed with X-tremeGENE siRNA transfection reagent (Roche Applied Science, Mannheim, Germany) according to manufacturer instructions.

## 1.12 ROS assessment

ROS levels were determined using an ROS assay kit (Beyotime Biotechnology Co., Ltd.). Briefly, fresh-frozen sections of mouse carotid arteries were obtained immediately after SDT treatment. The cryosections were stained with 10 µM 2′,7′-dichlorodihydrofluorescein diacetate (DCFH-DA) for 30 min at 37°C. The nuclei were counterstained with 4′,6-diamidino-2-phenylindole (DAPI) and the sections were visualized under a fluorescence microscope (Olympus, Tokyo, Japan) immediately. In vitro, ILMs were stained with 10 μM DCFH-DA for 20 min at 37°C, followed by fluorescence intensity analysis using a fluorescence microplate reader (Tecan Group Ltd.).

## 1.13 Intracellular lipid assessment

At 4 h after SDT, ILMs were incubated with 30 ng/mL oxidized low-density lipoprotein for 48 h, and after washing with PBS, the cells were stained with oil red O working solution. The staining reagent was then removed, and the cells were washed with [distilled](javascript:;) [water](javascript:;). The oil red O was extracted with 100% isopropanol with shaking for 15 min, followed by collection and analysis using a spectrophotometer (BioTek Instruments, Winooski, VT, USA) at 500 nm. Intracellular cholesterol levels were analysed using a cholesterol/cholesteryl ester assay kit (Abcam Inc., Cambridge, UK) according to manufacturer instructions.

## 1.14 Assessment of inflammatory cytokines

At 24 h after SDT, supernatant of ILM cultures from each group was collected, and levels of cytokines [interleukin (IL)-6, monocyte chemoattractant protein-1 (MCP-1) and tumour necrosis factor (TNF)-α] were detected using ELISA kits (IL-6 and TNF-α: Novus Biologicals LLC., Centennial, CO, USA; MCP-1: BOSTER Biological Technology Co., Ltd., Wuhan, China) according to manufacturer instructions.

## 1.15 Real-time monitoring of intracellular ROS

ILMs were preloaded with DVDMS and stained with DCFH-DA (10 μM) before ultrasonic irradiation. Fluorescence signals were acquired during ultrasonic irradiation, as reported previously [^7^](#_ENREF_7).

## 1.16 Assessment of Nrf2 localization

ILMs were fixed and labelled with an anti-Nrf2 antibody, followed by a tetramethylrhodamine-conjugated secondary antibody. The nucleus was visualized by DAPI staining, and images were obtained using a laser scanning confocal microscope (Carl Zeiss AG, Oberkochen, Germany).

**1.17 TUNEL assay**

The apoptosis in atherosclerotic plaques was detected by using in situ Cell Death Detection Kit (Roche Applied Science, Mannheim, Germany), which was known as TUNEL assay. All procedures have been done according to the manufacturer’s instructions.

## 1.18 Cell viability assay

Iron loaded macrophages cultured on 96 well plates were incubated with different concentrations of DVDMS (0.1–0.9 µM) in the dark at 37 °C in a humidified atmosphere containing 5% CO2. After 8 hours, the survival rate of the cells was measured by CCK8 assay according to the protocol.

## 1.19 Intracellular kinetics of DVDMS fluorescence

To investigate the intracellular kinetics of DVDMS fluorescence, ILMs were cultured on 96 well plates and incubated in the dark with 0.2 μM DVDMS at 37 °C in a humidified atmosphere containing 5% CO2 for different time intervals. Then cells were washed with PBS and analyzed by fluorescence microplate reader.

## 1.20 Bioinformatics analysis

Bioinformatics analysis was applied to obtain potential transcription factors that regulated the transcription of FPN1 gene (*Slc40a1*). First, *Slc40a1* gene promoter sequence was obtained. In order to derive more potential acting transcription factors, the sequences from the gene body upstream 2 kb to beginning region 1 kb of the *Slc40a1* gene were defined as the promoter region. Then, the R Package of Transcription Factor Binding Site (TFBSTools) and the Match^TM^ software were used to identify the potential transcription factor binding site (TFBS) of *Slc40a1*. In total, 106 transcription factors were found by TFBSTools and 212 transcription factors were gotten by Match^TM^. The consistent results of the two tools included four transcription factors named as NFE2L2, FOXO1, SOX17 and MAFB. Finally, a transcriptional landscape of mouse blood stem/progenitor cell (GSE81682) was obtained from the Gene Expression Omnibus (GEO) database. The expression values of these four transcription factors and *Slc40a1* gene were extracted and the Pearson correlation coefficients were calculated.

#
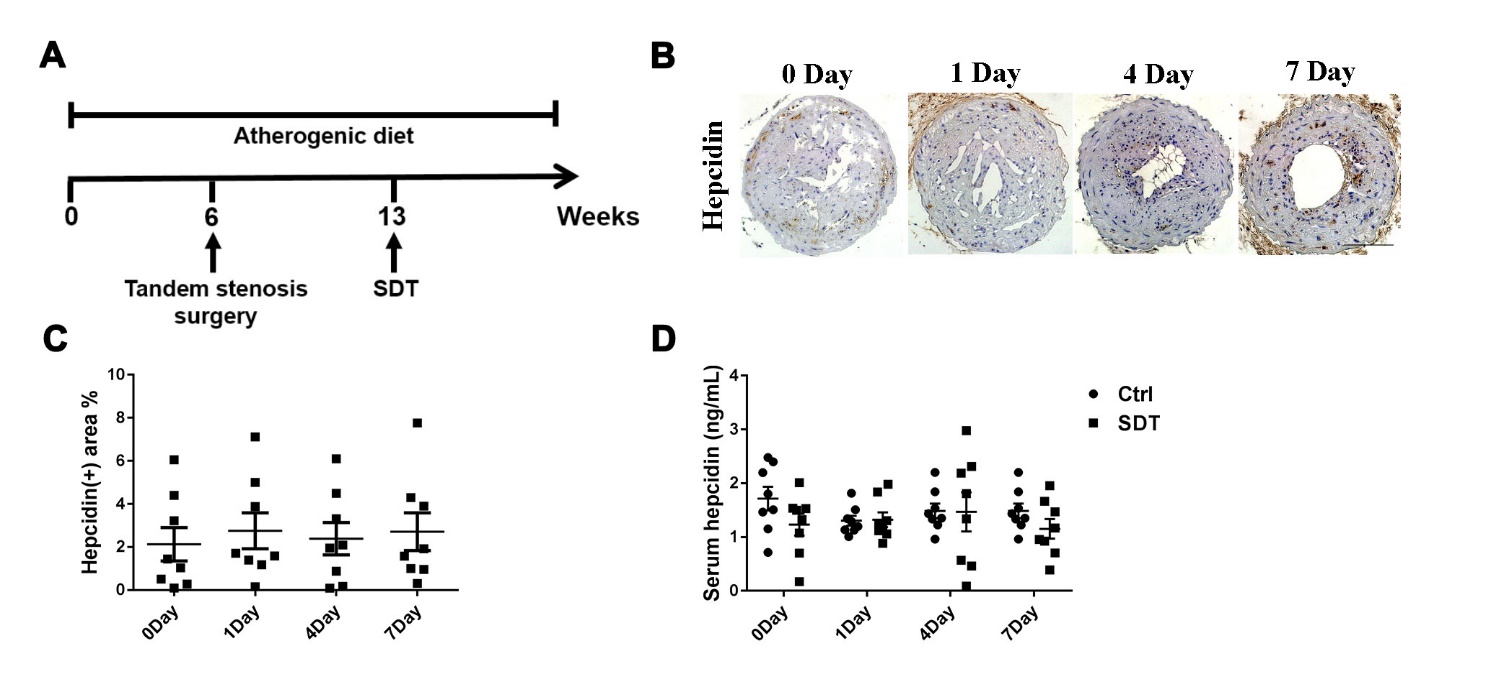
2. Supplementary Figures

**Figure S1. SDT has no effect on hepcidin expression in mice plaque or serum.** **(A)** Schematic diagram describing establishment of mice model and SDT treatment. **(B, C)** Immunohistochemistry (IHC) staining and relative quantification show SDT didn’t alter hepcidin expression in mouse plaques (*n* = 8/group). Scale bar, 50 μm. **(D)** SDT had no effect on serum hepcidin levels in mice (*n* = 8/group).


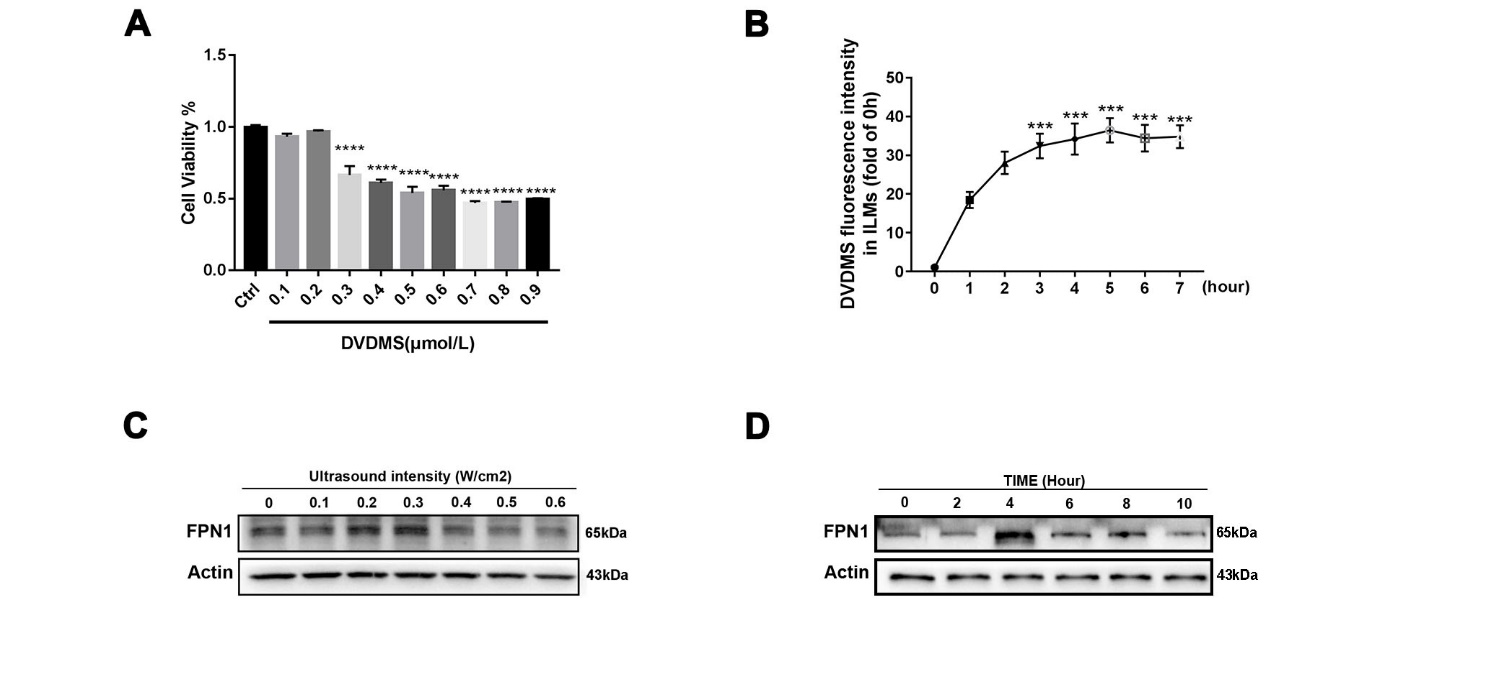
**Figure S2. Optimized parameters of SDT for ILMs. (A)** The survival rates of iron-loaded macrophages (ILMs) after sinoporphyrin sodium (DVDMS) incubation at different concentrations (*n* = 3/group). **(B)** Intracellular kinetics of DVDMS fluorescence in ILMs at different time points after incubating with 0.2 μM DVDMS (*n* = 3/group). **(C)** Western blot analysis shows that ferroportin 1 (FPN1) was highly expressed at the 0.2-0.3 W/cm^2^ ultrasound intensity. **(D)** Western blot analysis shows that ILMs had highest expression of FPN1 at 4 h after SDT. ****P* < 0.001, *****P* < 0.0001.

**
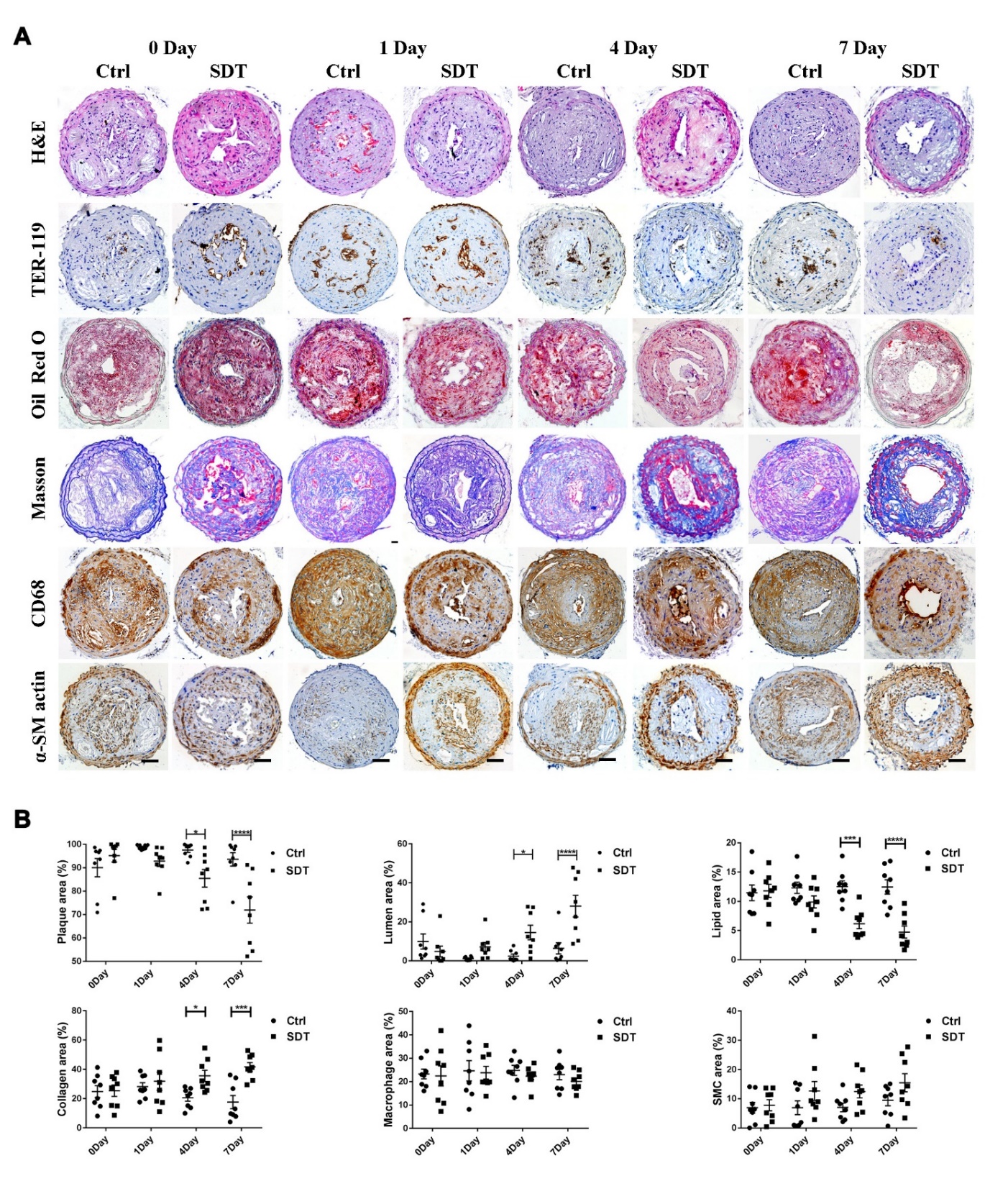
Figure S3.** **SDT exerts anti-atherosclerotic effect on ApoE^-/-^ mice plaque with high susceptibility of IPH.** **(A, B)** Histopathological staining and relative quantification shows that SDT induced size and composition changes in mice haemorrhagic plaques (n = 8/group). Scale bar, 50 μm. **P* ＜ 0.05, ****P* ＜ 0.001, *****P* ＜ 0.0001.

**
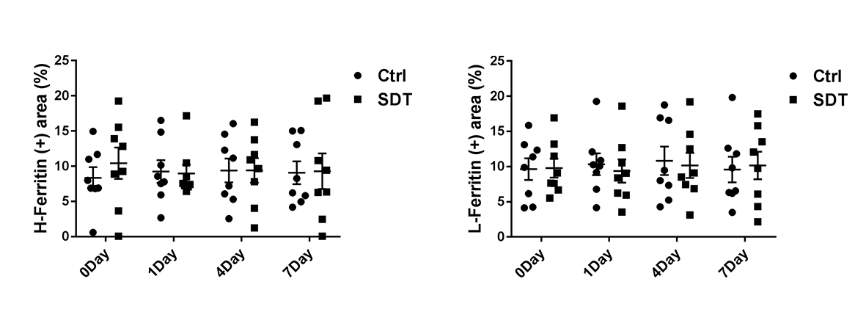
Figure S4. SDT has no effect on Ferritin expression in the media of mouse arteries.** Relative quantification show SDT didn’t alter H- and L-ferritin levels in media of mouse arteries (n = 8/group).


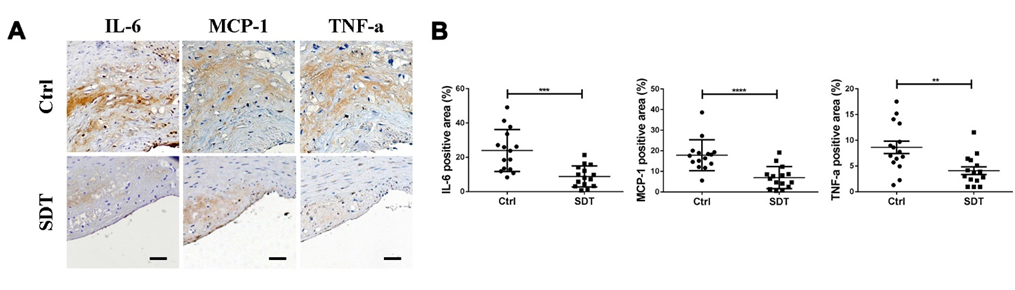


**Figure S5. SDT reduces inflammatory cytokines in rabbit plaque with IPH. (A, B)** IHC staining and relative quantification shows that SDT reduced interleukin-6 (IL-6), monocyte chemoattractant protein-1 (MCP-1) and tumor necrosis factor-α (TNF-α) levels in rabbit hemorrhagic plaque (*n* = 15/group). Scale bar, 50 μm. ***P* ＜ 0.01, ****P* ＜ 0.001, *****P* ＜ 0.0001.


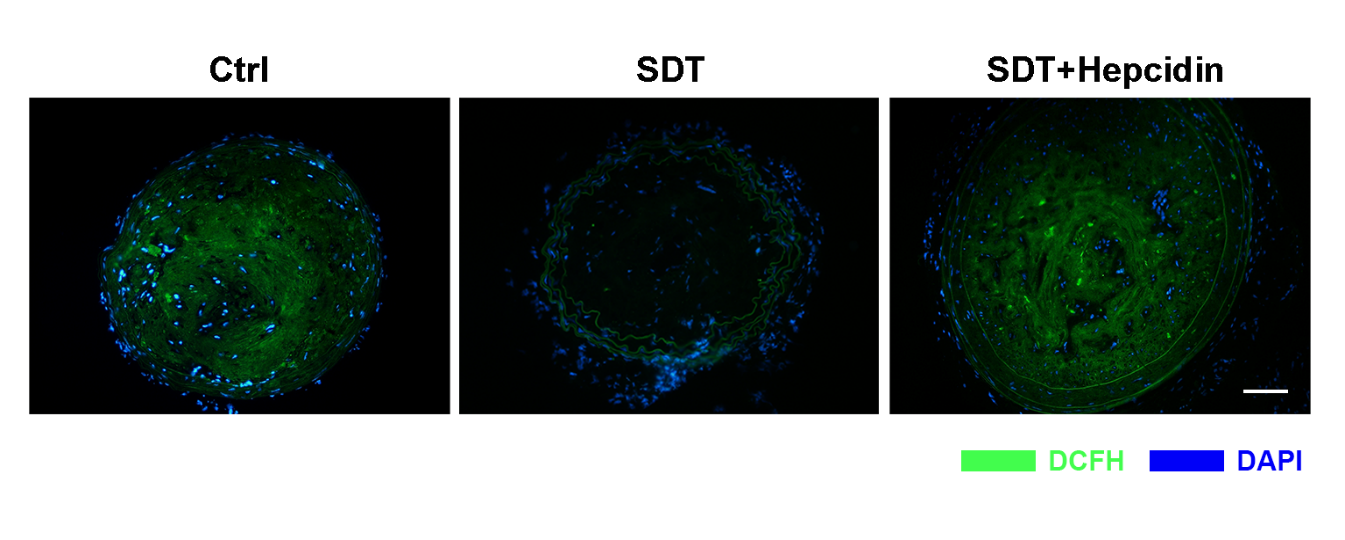
**Figure S6. SDT reduces Reactive oxygen species (ROS) levels in mouse plaque.** DCF fluorescence shows that the level of ROS within mouse plaque was decreased at day 7 after SDT, which was reversed by hepcidin treatment. Scale bar, 25 μm.

**
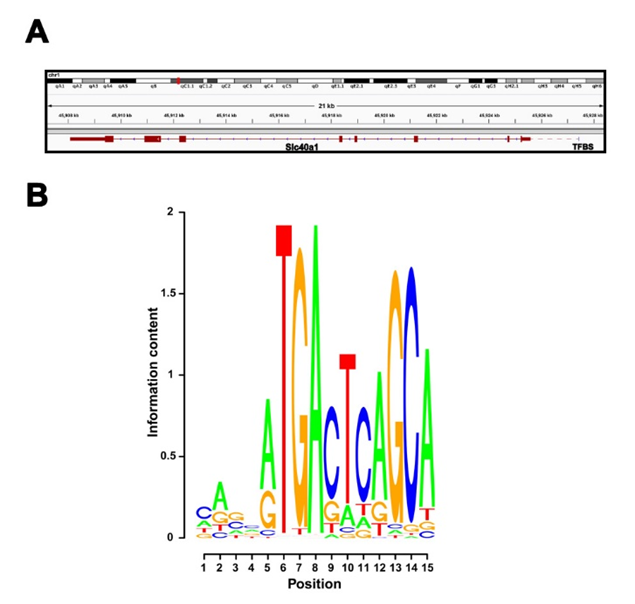
**

**Figure S7. Bioinformatics analysis results. (A)** Slc40a1 localization on chromosome 1, reference sequence and transcription factor binding site. **(B)** Functional domain prediction of Nfe2l2 (motif).


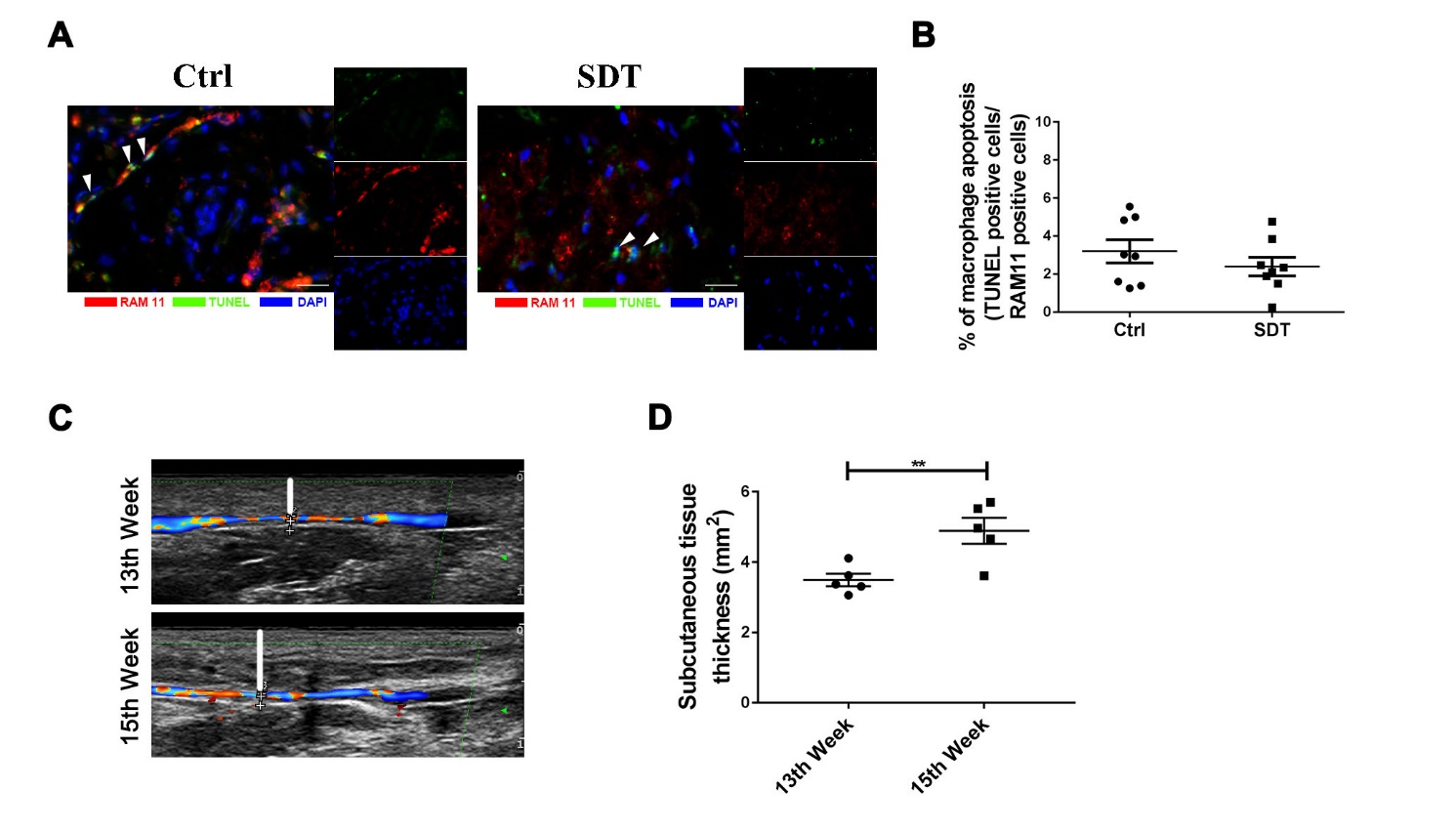


**Figure S8. SDT has no effect on macrophage apoptosis in rabbit plaque with IPH.** **(A, B)** TUNEL and immunofluorescence double staining and relative quantification show that SDT did not induce macrophage apoptosis in rabbit hemorrhagic plaque. Blue: nucleus; Red: macrophage; green: apoptosis; double red and green staining signifies the macrophages apoptosis. Arrowheads indicate positive double staining areas. (*n* = 8/group). Scale bar, 50 μm. **(C)** Ultrasonography images of rabbit lower limb with plaque model. The ultrasonography images were achieved at 13th week (before secondary surgical operation and erythrocytes injection) and 15th week (2 weeks after secondary surgical operation and erythrocytes injection). The white line indicates the subcutaneous tissue thickness from skin to artery. **(D)** Quantification of the subcutaneous tissue thickness of (C). n=5 for each group. ***P* ＜ 0.01.

**References**

1. Sun X, Guo S, Yao J, et al. Rapid inhibition of atherosclerotic plaque progression by sonodynamic therapy. *Cardiovasc Res*. 2019; 115(1): 190-203.

2. Kolodgie F D, Gold H K, Burke A P, et al. Intraplaque hemorrhage and progression of coronary atheroma. *N Engl J Med*. 2003; 349(24): 2316-2325.

3. Chen Y C, Bui A V, Diesch J, et al. A novel mouse model of atherosclerotic plaque instability for drug testing and mechanistic/therapeutic discoveries using gene and microRNA expression profiling. *Circ Res*. 2013; 113(3): 252-265.

4. Auriat A M, Silasi G, Wei Z, et al. Ferric iron chelation lowers brain iron levels after intracerebral hemorrhage in rats but does not improve outcome. *Exp Neurol*. 2012; 234(1): 136-143.

5. Kakhlon O and Cabantchik Z I. The labile iron pool: characterization, measurement, and participation in cellular processes. *Free Radic Biol Med*. 2002; 33(8): 1037-1046.

6. Wang H, Yang Y, Sun X, et al. Sonodynamic therapy-induced foam cells apoptosis activates the phagocytic PPARgamma-LXRalpha-ABCA1/ABCG1 pathway and promotes cholesterol efflux in advanced plaque. *Theranostics*. 2018; 8(18): 4969-4984.

7. Sun X, Xu H, Shen J, et al. Real-time detection of intracellular reactive oxygen species and mitochondrial membrane potential in THP-1 macrophages during ultrasonic irradiation for optimal sonodynamic therapy. *Ultrason Sonochem*. 2015; 227-14.
